# Factors Associated with Acceptance of Screening and Knowledge About Dementia in Older Adults in China: A Cross-Sectional Study

**DOI:** 10.3390/healthcare13131477

**Published:** 2025-06-20

**Authors:** Junli Wan, Dan Yang, Lining Xi, Huidan Yu, Xianwu Luo

**Affiliations:** School of Nursing, Wuhan University, 115 Donghu Road, Wuhan 430071, China; 2023283070028@whu.edu.cn (J.W.); 2023283030222@whu.edu.cn (D.Y.); liningxi@whu.edu.cn (L.X.); 00008456@whu.edu.cn (H.Y.)

**Keywords:** older adults, dementia screening, dementia knowledge, screening acceptance, nursing

## Abstract

**Background/Objectives**: Dementia is one of the leading causes of disability and dependence among older adults. Early screening may support timely intervention and risk management, contributing to better outcomes at the public health level. However, evidence relating to the factors influencing dementia screening acceptance and knowledge among older adults remains limited. This study aimed to assess dementia knowledge and screening acceptance among older adults, identify their associated factors, and explore the relationship between the two. **Methods**: A cross-sectional survey was conducted among 272 older adults in three Chinese communities. Data were collected using a self-administered questionnaire covering socio-demographic characteristics, dementia knowledge, and screening acceptance. The Dementia Knowledge Questionnaire and the Chinese version of the PRISM-PC scale were applied. Univariate and multivariate linear regression analyses were used. **Results**: The mean scores for dementia knowledge and screening acceptance were 18.86 ± 5.98 and 62.06 ± 22.18, respectively. Age and education level were negatively associated with screening acceptance. Women had higher knowledge scores than men. Income and social participation were positively associated with dementia knowledge. Knowledge level showed a weak positive correlation with screening acceptance. **Conclusions**: The study revealed that dementia knowledge and screening acceptance among older adults were moderate; nonetheless, both aspects warrant further improvement. Community-based efforts should prioritize health education, stigma reduction, and targeted interventions to enhance knowledge and promote proactive screening behavior.

## 1. Introduction

Dementia is a progressive neurodegenerative disease that severely impairs cognitive function, behavior, and memory and is one of the leading causes of disability and dependency among older adults worldwide [1]. Currently, there are about 57 million people living with dementia globally, with over 60% residing in low- and middle-income countries (LMICs) [1,2]. With improvements in education and better control of cardiovascular diseases, the incidence of dementia has declined in high-income countries; in contrast, LMICs are witnessing a continued rise in incidence due to accelerated population aging, urbanization, and lifestyle changes [2,3]. As the most populous developing country, China faces a particularly serious challenge—there are currently 15.33 million people with dementia, accounting for a quarter of the global total, and this number is expected to rise to 45.53 million by 2050 [4,5]. The economic burden of dementia is also substantial, with China’s annual expenditure reaching USD 167.74 billion, projected to soar to USD 1.89 trillion by 2050 [6]. In this context, dementia has become an urgent public health concern for China and a pressing challenge for global health systems.

Dementia currently lacks effective treatment and remains incurable; therefore, early screening and intervention are particularly crucial. Dementia screening generally refers to the preliminary assessment of individuals who may be in the early stages of dementia, using standardized cognitive evaluation tools to identify cognitive impairment [7,8]. Research has shown that nearly one third of dementia cases can be treated earlier if detected through timely screening [9], while delayed diagnosis not only postpones intervention but also significantly increases caregiving costs [10]. Alarmingly, more than half of dementia patients have never received a formal diagnosis [11], highlighting the insufficient coverage and delayed identification in primary care screening. Furthermore, dementia is a syndrome caused by diverse etiologies; screening facilitates early symptom detection and etiological clarification and supports personalized treatment [1,8]. Screening also helps to prevent the misdiagnosis of reversible conditions, thus improving diagnostic accuracy and clinical efficiency [7,8]. Therefore, dementia screening is not only of public health importance but also plays a pivotal role in clinical decision-making.

To proactively address the public health challenges posed by dementia, the Chinese government approved the Explore the Dementia Prevention and Treatment Specialty Services Work Program plan in 2020, which set a goal of raising the dementia screening rate among older adults in communities to 80% [12]. However, in real-world settings, the implementation of screening faces significant barriers: over 60% of patients live in communities that have never conducted such screenings, and 12.8% of older adults lack even a basic awareness of screening services [13]. Research has shown individuals’ knowledge of dementia directly influences their health-related behaviors; specifically, those with greater awareness are more likely to recognize early symptoms, seek professional help proactively, and participate in screenings [14]. However, the general public still lacks sufficient knowledge and understanding of dementia, which significantly hampers the achievement of high screening rates and timely diagnosis and intervention [4,8,15].

Moreover, the success of dementia screening programs largely depends on the degree to which older adults accept the screening process. Thus, it is crucial to scientifically assess the current status of screening acceptance among older adults and its influencing factors. Existing studies suggest that elderly individuals’ acceptance of dementia screening varies across countries [16,17,18]. For instance, a study in Germany found that 71.2% of older adults were willing to undergo regular dementia screening [16], whereas studies in other nations indicated substantially lower rates [17,18]. Factors such as age, education level, and personal beliefs have been identified as influencing screening acceptance [19,20]. While some studies suggest that gender and health status may play a role, others have reported no significant association with education level [18]. In addition, younger age, cohabitation with family members, literacy, and education level have been linked to increased dementia knowledge [15,21], though the existing evidence remains incomplete. Importantly, one study identified dementia-related knowledge as a significant predictor of an individual’s intention to undergo screening [22]. Thus, assessing both the level of dementia knowledge and screening acceptance among older adults is critical. However, few studies have examined the influencing factors of these variables, and even fewer have explored the association between them. This study aimed to assess the acceptance of dementia screening and the level of dementia-related knowledge among community-dwelling older adults, as well as to examine the relationship between the two. The study further explored key demographic and social factors influencing acceptance and knowledge, in order to inform the development of effective intervention strategies.

Research questions:

RQ1: What are the levels of dementia screening acceptance and dementia-related knowledge among community-dwelling older adults?

RQ2: What factors influence older adults’ attitudes toward dementia screening and their level of dementia-related knowledge?

RQ3: Is there a statistically significant correlation between dementia-related knowledge and acceptance of dementia screening?

## 2. Materials and Methods

### 2.1. Study Design

A cross-sectional study was conducted in Wuhan, China, between November 2020 and April 2021. Ethical approval was obtained from the Ethics Committee of the School of Nursing, Wuhan University (Approval No. 2020YF0009).

### 2.2. Participants

We employed a convenience sampling method and recruited older adults residing in three communities in Wuhan: Tangjiadun Community Health Service Center in Jianghan District, Shuiguohu Community Health Service Center in Wuchang District, and Hanzheng Street Community Health Service Center in Qiaokou District, Hubei Province. Invitations were distributed through these community health centers using printed leaflets, which clearly stated that participation in the study was entirely voluntary.

According to the literature [23], the recommended sample size for multiple linear regression analysis is at least 10 times the number of independent variables. This study included 17 variables, and based on the recommendation that the sample size for multiple linear regression should be at least 10 times the number of independent variables, the minimum required sample size was estimated to be 170. To account for a potential 20.0% rate of invalid or incomplete responses, the adjusted required sample size was approximately 213. However, a total of 280 participants were ultimately recruited and included in the final analysis. This was carried out to enhance the statistical power of the study, ensure greater robustness in the findings, and improve the representativeness of the sample. The inclusion criteria for the participants were as follows: (1) age ≥ 60 years; (2) residing within the community for at least 6 months; and (3) the ability to communicate effectively. The exclusion criteria included (1) a history of acute cerebrovascular disease within the last 3 months; (2) serious or unstable medical conditions affecting cognitive assessment, such as severe heart, liver, or lung diseases; (3) individuals diagnosed with malignant tumors; (4) individuals with active epilepsy; (5) individuals with severe mental disorders, such as schizophrenia; (6) individuals with advanced cognitive impairment who were unable to comprehend or independently complete the questionnaire; and (7) individuals diagnosed with neurodegenerative diseases primarily characterized by motor symptoms (e.g., Parkinson’s disease, Parkinson’s disease dementia, dementia with Lewy bodies), as these conditions have distinct clinical trajectories and may confound the assessment of dementia-related knowledge and screening acceptance.

### 2.3. Measurement Tools

A self-administered questionnaire including socio-demographic characteristics, a self-developed Dementia Knowledge Questionnaire, and the Chinese version of the Perceptions Regarding Investigational Screening for Memory in Primary Care (PRISM-PC) scale were used as the measurement tools.

The socio-demographic characteristics included age, gender, education level, housing status, monthly income, chronic illness, participation in social activities, and exercise.

Based on a review of the relevant literature [15,21,24], the researchers developed the initial version of the Dementia Knowledge Questionnaire and consulted five experts in neurology, general medicine, and sociology to evaluate its content. The content validity index (CVI) of the questionnaire was 0.95, indicating strong content validity. The final questionnaire consisted of 26 items covering five domains: risk factors, early symptoms, disease progression, treatment effects, and preventive measures related to dementia. Each item was scored on a binary scale: “agree” was scored as 1, and “disagree” as 0. A higher total score reflected a higher level of dementia-related knowledge.

The PRISM-PC scale was developed to measure acceptance and attitudes toward dementia screening [25]. It includes 29 items in two dimensions: the acceptance of screening and perceptions of the harms and benefits of dementia screening. The Chinese version was introduced and translated by Ji et al. [26]. A five-point Likert scale was used for scoring (one point for ‘totally disagree’ to five for ‘totally agree’). All items were scored using the reverse scoring method. The following conversion formula was used: Conversion score = (actual score − lowest possible score for this dimension)/difference between highest possible score and lowest possible score for this dimension ×100 to convert the score for each dimension from 0 to 100, with a higher conversion score indicating a higher degree of agreement with the items contained in the dimension. The Cronbach’s α of the Chinese version was 0.876 [26].

### 2.4. Data Collection and Quality Control

The research team conducted on-site data collection at three community health service centers, all of which received institutional approval. Data collection was carried out by one graduate and three undergraduate nursing students, all of whom received standardized training to ensure procedural consistency and data quality. A face-to-face questionnaire survey was used in this study. Prior to the formal survey, investigators used standardized instructions to explain the purpose and method of completing the questionnaire, and participants completed it independently after giving informed consent. For participants unable to complete the questionnaire on their own, the researchers patiently read the questions aloud in a neutral and non-suggestive manner, ensuring comprehension before recording responses on their behalf. Returned questionnaires showing patterns such as repeated answers, identical choices, or missing data were deemed invalid and excluded.

### 2.5. Statistical Analyses

Data analysis was performed using SPSS version 23.0 [27]. Descriptive statistics were used to summarize participants’ characteristics, presented as frequencies and percentages. Group differences were examined using one-way ANOVA and independent samples *t*-tests. Multivariate linear regression analyses were performed using a stepwise approach, adjusting for potential confounders to assess the association between predictor variables and scores on both the acceptance dimension and the Dementia Knowledge Questionnaire. Categorical variables, including education level, housing status, and participation in social activities, were converted into dummy variables and included in the regression models. Missing data were handled using multiple imputation with mean substitution. Pearson’s correlation analysis was conducted to assess the relationship between dementia knowledge and screening acceptance. A two-tailed *p*-value of <0.05 was considered statistically significant.

## 3. Results

### 3.1. Socio-Demographic Characteristics

A total of 280 questionnaires were distributed in this study. After excluding 4 incomplete questionnaires, 2 with patterned responses, and 2 with identical choices throughout, 272 valid questionnaires were retained, yielding a valid response rate of 97.1%.

In this study, 54.8% of the participants were male, with a mean age of 71.83 ± 7.39 years. The majority were under the age of 80, and approximately two thirds had attained at least a high school education. About half of the respondents lived with others, and most reported having at least one chronic illness (75.4%). Participation in social activities was relatively evenly distributed across different frequencies, with each category accounting for approximately one quarter of responses. In terms of physical activity, the majority of participants reported exercising regularly, with more than one third engaging in exercise more than three times per week (see Table 1).

### 3.2. Factors Associated with Acceptance of Dementia

The mean score for acceptance of dementia was 62.06 ± 22.18, indicating that people had a positive attitude toward accepting dementia screening. In the univariate analysis, education level and monthly income were statistically significant. After adjustment, age (60–69: adjusted mean difference 9.66, 95%CI [2.05 to 17.28]; 70–79: adjusted mean difference 6.16, 95%CI [−1.26 to 13.58]) and education level (primary school and below: adjusted mean difference 6.61, 95%CI [−2.61 to 15.83], junior school: adjusted mean difference 10.79, 95%CI [0.97 to 20.62], high school or junior college: adjusted mean difference 1.69, 95%CI [−7.10 to 10.49]) had an impact on the acceptance of dementia screening in older adults, *p* < 0.05. Older adults aged from 60 to 69 and those with junior school degrees had the highest acceptance of dementia screening scores. Details are shown in Table 2.

### 3.3. Factors Associated with Dementia Knowledge Questionnaire

The mean score for dementia knowledge was 18.86 ± 5.98. Participants scored highest on the items “Dementia can be detected and diagnosed early” and “Steps can be taken to delay or prevent the onset of dementia,” with mean scores of 0.88 ± 0.33 and 0.84 ± 0.37, respectively. In contrast, the lowest score was recorded for the item “Prevention of dementia starts in early youth” (0.26 ± 0.44). Detailed item-level results are presented in Table 3.

As shown in Table 4, in the univariate analysis, income, participation in social activities, and exercise were statistically significant. After adjustment, gender (adjusted mean difference (−1.50, 95% CI [−2.88 to −0.12]), income (<1000 RMB: adjusted mean difference −1.30, 95% CI [−3.75 to 1.14], 1000–2999 RMB: adjusted mean difference 1.85, 95% CI [−0.44 to 4.15], 3000–4999 RMB: adjusted mean difference 1.23, 95% CI [−0.91 to 3.37]), and participation in social activities (never: adjusted mean difference −3.97, 95% CI [−6.12 to −1.82], once or twice a month: adjusted mean difference −4.12, 95% CI [−5.91 to −2.34], three to five times a month: adjusted mean difference −3.36, 95% CI [−5.25 to−1.48]) had an impact on the level of knowledge on dementia in older adults. Older adults with an income from RMB 3000 to 4999 and participation in social activities more than five times a month had the highest scores on knowledge of dementia.

The adjusted R^2^ of screening attitudes was 0.26 and the adjusted R^2^ of knowledge of dementia was 0.16. Pearson correlation coefficient analysis showed that the r value was 0.14 (*p* = 0.020), indicating that there was a weak positive correlation between dementia knowledge and acceptance of screening attitudes.

## 4. Discussion

This study aimed to investigate older adults’ acceptance of dementia screening and their level of dementia-related knowledge and to analyze the influencing factors and their interrelationship. The findings indicated that the respondents generally held a positive attitude toward dementia screening, and their dementia knowledge was at a moderate level. Furthermore, the study found a weak positive correlation between dementia knowledge and screening acceptance, suggesting that improving dementia knowledge among older adults might have facilitated their willingness to undergo screening.

This study found that the acceptance of dementia screening among older adults was moderate, with an average acceptance score of 62.06 ± 22.18. This acceptance level is consistent with findings from a German study [16], but contrasts with results reported in Japan and the United States [18,28]. These discrepancies may reflect variations in national primary healthcare systems and cultural perceptions [9,17]. The relatively low acceptance of dementia screening among community-dwelling older adults may stem from several factors. Firstly, many older adults perceive dementia as a normal part of aging rather than as a medical condition [29]. Additionally, receiving a dementia diagnosis may expose them to social discrimination, thereby exacerbating the stigma surrounding the condition [30].

The study showed that age and educational level had a significant impact on the acceptance of dementia screening. Younger older adults showed a higher acceptance of dementia screening, aligning with previous research findings [18,19,21]. Possible explanations include younger seniors tending to have more active thinking, greater openness to new knowledge, increased resilience to illness, and a higher willingness to receive dementia-related information [9,31,32]. Conversely, declines in memory, hearing, and vision among older individuals may limit their ability to acquire dementia knowledge. Interestingly, older adults with lower education levels exhibited higher acceptance than those with higher education, differing from findings of previous studies [15,33,34]. The reasons behind this discrepancy remain unclear but may be related to higher education levels being associated with greater sensitivity to stigma surrounding dementia [15,35]. Future interventions should therefore adopt age-specific strategies, such as employing more interactive and accessible education programs tailored to older populations. Additionally, efforts to reduce dementia-related stigma, particularly among highly educated older adults, are essential. Further studies could explore targeted educational methods and anti-stigma interventions to clarify the underlying factors and improve dementia screening acceptance.

Regarding dementia knowledge, our study found that dementia knowledge among older adults was at an intermediate level. High scores were observed in items related to the early detection and diagnosis of dementia, as well as measures to delay or prevent its onset, suggesting that community-dwelling older adults have good awareness of early screening and preventive interventions for dementia. However, the relatively low scores on items regarding the risk factors of dementia and the importance of initiating preventive measures during early adulthood highlight the need for enhancing older adults’ understanding of these aspects. Additionally, the results indicated that gender played a significant role, with women exhibiting higher levels of knowledge than men. This finding is consistent with previous studies suggesting that women are generally more interested in, and better informed about, health-related issues than men [9,21]. Income also showed a positive association with dementia knowledge, likely because individuals with higher socioeconomic status have greater access to educational resources and health-related information [30]. Furthermore, participation in social activities was positively linked to dementia knowledge. Social engagement may enhance cognitive functioning and create opportunities for information exchange, thus helping older adults gain a better understanding of dementia [36,37,38].

This study further found a weak positive correlation between dementia knowledge and screening acceptance (r = 0.14, *p* = 0.020), suggesting that improving dementia knowledge among older adults might have helped enhance their willingness to accept screening. According to the Knowledge, Attitudes, Beliefs, and Practices (KABP/KAP) model, knowledge serves as the foundation for shaping beliefs and attitudes; only when individuals possess adequate knowledge of relevant health issues can they form accurate perceptions and develop health-promoting behaviors while modifying harmful ones [30]. Although many participants reported being aware of dementia as a disease, only a few believed they could distinguish early symptoms of dementia from normal aging. Additionally, more than half of the participants had a limited understanding of the risk and protective factors associated with dementia [39].

### 4.1. Implications for Policy and Practice

Although this study demonstrated that most community-dwelling older adults held a positive attitude toward dementia screening, the actual coverage rate in China remains below 60% [13], indicating a clear gap between knowledge and practice. This gap not only stems from individuals’ limited understanding of dementia and insufficient motivation to undergo screening but also reflects deeper systemic barriers, particularly within the primary healthcare system [29]. First, current primary healthcare institutions face multiple challenges in the implementation of dementia screening. Many primary care physicians have a limited ability to identify dementia, lack systematic training, and are not proficient in using cognitive screening tools [4]; some even hold misconceptions such as “screening is useless” or “intervention is impossible” [40]. These issues not only restrict the implementation of screening but also undermine older adults’ confidence and willingness to participate. Therefore, promoting early detection and intervention for dementia requires not only enhancing older adults’ knowledge but also strengthening education and capacity-building for frontline healthcare providers. Specifically, government and health authorities could consider integrating dementia screening into the continuing education system for primary healthcare workers, establishing a standardized training mechanism across urban and rural areas to ensure their proficiency in practical screening tools and communication strategies [4]. In public education, efforts should be focused on older adults with low health literacy, using visually rich and plain-language materials to disseminate dementia knowledge, especially regarding risk factors, modifiability, and early identification.

### 4.2. Limitations

This study has several limitations. First, due to its cross-sectional design, causal relationships between dementia knowledge and screening acceptance cannot be established. Second, the data were self-reported, which may introduce recall or social desirability bias. Third, the study sample was drawn exclusively from three communities in Wuhan. Although these communities represent, to some extent, diverse demographic and socioeconomic backgrounds, the findings may not be generalizable to older adult populations in other regions or cultural contexts. Fourth, the educational level of our participants was relatively high, with two thirds having completed at least high school, which may not fully reflect the educational distribution of the general older adult population in China. This could limit the generalizability of our findings. Future research should adopt longitudinal designs, recruit more diverse and geographically dispersed samples, and consider incorporating objective measures of dementia knowledge and screening behaviors. These steps will help to enhance the robustness and external validity of the findings and provide deeper insights into the mechanisms underlying screening acceptance.

## 5. Conclusions

This study revealed that community-dwelling older adults had a moderate level of acceptance of dementia screening, and their dementia-related knowledge was also at a moderate level, with a weak positive correlation between the two. Age, educational level, gender, income, and participation in social activities significantly influenced their knowledge and attitudes. Acceptance was significantly influenced by age and education, while knowledge level was associated with gender, income, and social engagement. The findings suggest that enhancing older adults’ understanding of dementia risk factors and early symptoms and reducing stigma may effectively improve their willingness to undergo screening and promote early detection and intervention.

## Figures and Tables

**Table 1:** Demographic characteristics of participants (*n* = 272).

Participant Characteristics	*N* (%)
Gender	
Male	149 (54.80)
Female	123 (45.20)
Age (years)	
60–69	115 (42.30)
70–79	110 (40.40)
≥80	47 (17.30)
Education level	
Primary school and below	47 (17.30)
Junior school	49 (18.00)
High school or junior college	120 (44.10)
Bachelor’s degree or above	56 (20.60)
Housing status	
Living alone	46 (16.90)
Living with someone	128 (47.10)
Living in an older adult care facility	98 (36.00)
Income	
<1000 RMB	86 (31.60)
1000–2999 RMB	65 (23.90)
3000–4999 RMB	54 (19.90)
≥5000 RMB	67 (24.60)
Chronic illness	
Yes	205 (75.40)
No	67 (24.60)
Participation in social activities	
Never	58 (21.30)
Once or twice a month	91 (33.50)
Three to five times a month	55 (20.20)
More than five times a month	68 (25.00)
Exercise	
Never	35 (12.90)
Once a week	69 (25.40)
Two or three times a week	64 (23.50)
More than three times a week	104 (38.20)

**Table 2:** Difference in acceptance of dementia screening scores (*n* = 272).

Variables	Mean (SD)	Unadjusted Mean Difference (95% CI)	Unadjusted *p*-Value	Adjusted Mean Difference (95% CI) ^d^	Adjusted *p*-Value ^c^
Gender					
Male	62.41 (21.50)	0.80 (−4.53, 6.12)	0.769 ^a^	2.97 (−2.54, 8.48)	0.291
Female	61.62 (23.08)	Reference		Reference	
Age (years)					
60–69	64.67 (21.22)	5.94 (−1.87, 13.76)	0.066 ^b^	9.66 (2.05, 17.28)	0.045
70–79	61.55 (22.50)	2.82 (−5.03, 10.69)		6.16 (−1.26, 13.58)	
≥80	58.73 (22.78)	Reference		Reference	
Education level					
Primary school and below	63.99 (22.59)	8.19 (1.22, 15.16)	0.012 ^b^	6.61 (−2.61, 15.83)	0.048
Junior school	68.52 (18.61)	12.72 (4.21, 21.24)		10.79 (0.97, 20.62)	
High school or junior college	58.25 (24.33)	2.44 (−5.97, 10.87)		1.69 (−7.10, 10.49)	
Bachelor’s degree or above	55.80 (20.45)	Reference		Reference	
Housing status					
Living alone	65.40 (21.75)	4.75 (−2.72, 12.23)	0.155 ^b^	2.24 (−5.57, 10.06)	0.085
Living with someone	60.64 (23.39)	−0.69 (−6.62, 5.24)		−4.71 (−10.65, 1.23)	
Living in an older adult care facility	61.34 (20.50)	Reference		Reference	
Income					
<1000 RMB	63.23 (20.63)	6.95 (−0.11, 14.00)	0.048 ^b^	2.37 (−7.43, 12.17)	0.676
1000–2999 RMB	66.86 (22.29)	10.58 (3.04, 18.11)		5.34 (−3.88, 14.56)	
3000–4999 RMB	61.57 (22.95)	5.29 (−2.62, 13.21)		0.76 (−7.82, 9.33)	
≥5000 RMB	56.28 (22.59)	Reference		Reference	
Chronic illness					
Yes	62.62 (21.25)	2.29 (−3.85, 8.45)	0.463 ^a^	3.01 (−3.04, 9.05)	0.302
No	60.32 (24.93)	Reference		Reference	
Participation in social activities					
Never	65.23 (23.12)	6.10 (−1.70, 13.90)	0.336 ^b^	4.02 (−4,61, 12.65)	0.366
Once or twice a month	60.71 (22.37)	1.58 (−5.41, 8.58)		−0.38 (−7.53, 6.78)	
Three to five times a month	64.55 (21.57)	5.42 (−2.50, 13.33)		5.61 (−1.96, 13.18)	
More than five times a month	59.13 (22.19)	Reference		Reference	
Exercise					
Never	63.81 (24.27)	4.91 (−3.60, 13.44)	0.218 ^b^	0.90 (−8.72, 10.52)	0.438
Once a week	65.57 (17.49)	6.68 (−0.08, 13.46)		5.17 (−2.01, 12.34)	
Two or three times a week	62.43 (22.18)	3.54 (−3.39, 10.47)		2.73 (−3.99, 9.45)	
More than three times a week	58.89 (24.05)	Reference		Reference	

SD, standard deviation; CI, confidence interval; ANOVA, analysis of variance; RMB, Renminbi. ^a^ *p*-values based on independent *t*-test; ^b^ *p*-value based on ANOVA test; ^c^ *p*-value based on generalized linear regression model. ^d^ Estimated from a multiple linear regression model with acceptance score as the dependent variable and all other factors as covariates. Adjusted mean differences represent beta coefficients.

**Table 3:** Dementia Knowledge Questionnaire scores of older adults (*n* = 272).

Items	Mean ± Standard Deviation
Dementia is not a disease, it’s a natural aging phenomenon	0.47 ± 0.50
Dementia can be detected and diagnosed early	0.88 ± 0.33
Steps can be taken to delay or prevent the onset of dementia	0.84 ± 0.37
There is currently no specific treatment for dementia	0.70 ± 0.46
Prevention of dementia starts in early youth	0.26 ± 0.44
Risk Factors for dementia	0.52 ± 0.31
Early Manifestations of dementia	0.76 ± 0.29

**Table 4:** Difference in Dementia Knowledge Questionnaire scores (*n* = 272).

Variables	Mean (SD)	Unadjusted Mean Difference (95% CI)	Unadjusted *p*-Value	Adjusted Mean Difference (95% CI) ^d^	Adjusted *p*-Value ^c^
Gender					
Male	18.58 (5.99)	−0.60 (−2.04, 0.83)	0.409 ^a^	−1.50 (−2.88, −0.12)	0.033
Female	19.19 (5.97)	Reference		Reference	
Age (years)					
60–69	19.41 (5.96)	−0.33 (−2.45, 1.79)	0.207 ^b^	0.85 (−1.05, 2.75)	0.108
70–79	17.93 (17.93)	−1.81 (−3.94, 0.32)		−0.69 (−2.54, 1.16)	
≥80	19.74 (5.43)	Reference		Reference	
Education level					
Primary school and below	17.93 (6.94)	−1.00 (−2.90, 0.89)	0.090 ^b^	−1.95 (−4.25, 0.35)	0.292
Junior school	19.72 (5.26)	0.79 (−1.52, 3.11)		−0.62 (−3.08, 1.84)	
High school or junior college	20.22 (5.37)	1.30 (−0.99, 3.59)		−0.63 (−2.83, 1.58)	
Bachelor’s degree or above	18.93 (4.42)	Reference		Reference	
Housing status					
Living alone	20.50 (5.10)	1.62 (−0.49, 3.73)	0.111 ^b^	1.19 (−0.76, 3.14)	0.280
Living with someone	18.16 (6.57)	−0.73 (−2.32, 0.87)		−0.035 (−1.83, 1.13)	
Living in an older adult care facility	18.88 (5.46)	Reference		Reference	
Income					
RMB < 1000	16.81 (7.50)	−1.72 (−4.23, 0.79)	<0.001 ^b^	−1.30 (−3.75, 1.14)	0.003
RMB 1000–2999	20.46 (4.77)	1.92 (−0.76, 4.61)		1.85 (−0.44, 4.15)	
RMB 3000–4999	20.57 (4.68)	2.04 (−0.78, 4.85)		1.23 (−0.91, 3.37)	
RMB ≥ 5000	18.54 (4.94)	Reference		Reference	
Chronic illness					
Yes	19.20 (6.13)	1.41 (−0.14, 2.97)	0.093 ^a^	1.23 (−0.27, 2.74)	0.109
No	17.79 (5.40)	Reference		Reference	
Participation in social activities					
Never	15.53 (5.98)	−5.61 (−8.30, −2.92)	<0.001 ^b^	−3.97 (−6.12, −1.82)	<0.001
Once or twice a month	19.18 (5.65)	−4.16 (−6.57, −1.75)		−4.12 (−5.91, −2.34)	
Three to five times a month	19.69 (4.88)	−3.65 (−6.38, −0.93)		−3.36 (−5.25, −1.48)	
More than five times a month	21.14 (4.67)	Reference		Reference	
Exercise					
Never	21.66 (5.53)	3.07 (0.79, 5.35)	0.020 ^b^	2.31 (−0.09, 4.70)	0.066
Once a week	18.51 (5.67)	−0.08 (−1.89, 1.73)		−0.68 (−2.47, 1.11)	
Two or three times a week	18.14 (6.06)	−0.45 (−2.30, 1.40)		−0.91 (−2.59, 0.77)	
More than three times a week	18.59 (6.09)	Reference		Reference	

SD, standard deviation; CI, confidence interval; ANOVA, analysis of variance; RMB, Renminbi. ^a^ *p*-values based on independent *t*-test. ^b^ *p*-value based on ANOVA test. ^c^ *p*-value based on linear regression model. ^d^ Estimated from a multiple linear regression model with the acceptance score as the dependent variable and all other factors as covariates. Adjusted mean differences represent beta coefficients.

## Data Availability

The raw data supporting the conclusions of this article will be made available by the authors on request.
